# Effect of *Moringa Oleifera* leaf powder supplementation on reducing anemia in children below two years in Kisarawe District, Tanzania

**DOI:** 10.1002/fsn3.1110

**Published:** 2019-07-04

**Authors:** Angela E. Shija, Susan F. Rumisha, Ndekya M. Oriyo, Stella P. Kilima, Julius J. Massaga

**Affiliations:** ^1^ National Institute for Medical Research Da‐es‐salaam Tanzania

**Keywords:** anemia, children below 2 years, *Moringa oleifera*, supplementation

## Abstract

Anemia is a nutritional disorder that affects mostly children below 2 years and is mainly contributed by iron deficiency. *Moringa oleifera* leaves are rich in iron and other essential nutrients necessary for iron metabolism. We investigated the effect of *M. oleifera* leaf powder supplementation on reducing anemia among children below 2 years. A community‐based interventional study was conducted that enrolled 95 anemic children who were followed for 6 months. The intervention communities received *M. oleifera* leaf powder and nutrition education, while control communities only received nutrition education. Changes on mean hemoglobin (Hb) concentration and anemia prevalence were compared between the two groups using *t* test and proportional test where appropriate. At baseline, the mean Hb concentrations of control and intervention groups were 7.9 g/dl  (*SD* = 1.3) and 8.3 g/dl (*SD* = 1.6) g/L, respectively (*p*‐value = 0.0943). After 6 months, anemia prevalence significantly decreased in the intervention group by 53.6% (100%–46.4%; *p* < 0.001) compared to 13.6% (100%–86.4%; *p* = 0.005) in control community. The mean Hb was 10.9 g/dl (95% CI: 10.2–11.4) for intervention and 9.4 g/dl (95% 7.8–10.1) for control (*p*‐value = 0.002). The effect was also observed in the reduction of the prevalence of moderate and severe anemia in the intervention communities by 68.2% and 77.9%, respectively, and by 23.3% and 56.9%, respectively, in the control communities. Increasing amount and time of using *M. oleifera* supplementation resulted to significant reduction in anemia cases therefore can be used as complementary solution in addressing anemia among children especially when the use of infant formulas and fortified food product is very poor.

## 1 INTRODUCTION

High anemia prevalence in children below 2 years is a public health concern in Tanzania. Recent national surveys indicate that more than three‐quarters in children aged 6–24 months have been affected by anemia in the past decade (MoHCDGEC, MoH, NBS, OCGS, & ICF, 2016; NBS & ICF Macro, 2011). Children in this age group are at a critical period of rapid growth and development that increases the demand for iron and other nutrients (Domellöf et al., 2014). Approximately two‐thirds of total body iron is used in the formation of hemoglobin; hence, its deficiency becomes the main contributing factor to anemia (Baker & Greer, 2010; Caulfield, Richard, Rivera, Musgrove, & Black, 2006; Stoltzfus & Dreyfuss, 1998; WHO, 2008c, 2011a, 2011b, 2014). Iron deficiency in this period of life affects brain development and may have irreversible impact on cognitive development (Caulfield et al., 2006; Ewusie, Ahiadeke, Beyene, & Hamid, 2014; FAO & WHO, 2001; Freire, Kahn, McGuire, & Post, 2003; Thompson, 2011; WHO & FAO, 2006), which negatively affects future learning and earning capabilities (Tulchinsky, 2010). Iron deficiency anemia (IDA) is estimated to account for 40%‐60% of mental impairment in children predominantly 6–24 months from low‐income countries (MI & UNICEF, 2004). Moreover, IDA lowers the body’s capacity to fight against diseases (Domellöf et al., 2014; Thompson, 2011; WHO, 2014), which may lead to frequent illness that affects a child’s overall health and development (Caulfield et al., 2006; Gorstein, Sullivan, Parvanta, & Begin, 2007; WHO, 2001, 2008b). Common methods used to fight against nutritional anemia include iron supplementation and food‐based approaches. Iron supplements are commonly distributed in the form of highly concentrated tablets or syrup, consisting of a single or multiple nutrients, to the highly affected individuals or high‐risk groups as a quick and short‐term solution to IDA (FAO & CAB International, 2010; WHO, 2001; WHO & FAO, 2006). Food‐based approaches include food fortification, use of multiple micronutrient powders (commonly known as “sprinkles”), and dietary diversification (FAO, 2014; WHO, 2008c, 2014; WHO & FAO, 2006). The majority of families, especially those in rural communities, consume most of their food directly from their own farms. Therefore, dietary diversification through promoting locally available food products with high nutrient density continues to be a long‐term, cost‐effective, and sustainable solution to iron deficiency (Caulfield et al., 2006; MI, 2009; WHO, 2008c; WHO & FAO, 2006).

*Moringa oleifera* leaf powder has been found to have most of the essential nutrients required for good health (Ashifaq, Basra, & Ashifaq, 2012; Beth & Lindsay, 2005; Hiawatha Bey, 2010; Yang et al., 2006). The leaf powder is rich in multiple mineral and vitamins including iron, vitamin A (carotenoid), and vitamin C which are important for iron metabolism. In addition, *Moringa* has an added advantage in solving multiple malnutrition problems since it is rich in all essential amino acids, which are building blocks for proteins that are necessary for cell growth (Ashifaq et al., 2012; Busani, Patrick, Arnold, & Voster, 2011; Foidl, Makkar, & Becker, 2001). Studies conducted in different countries such as Senegal and India; the use of *Moringa* was reported to reduce malnutrition in children as well as vitamin A and protein deficiencies (Fahey, 2005; Fuglie, 2001; Mahmood, Mugal, & Haq, 2010; Srikanth, 2014). Safety evaluation studies on *Moringa* have shown no toxicity when consumed in large quantities (Devaraj, Asad, & Prasad, 2007; Luqman, Srivastava, Kumar, Maurya, & Chanda, 2012; Stohs & Hartman, 2015), with no adverse side effects reported by those who used it as part of their daily meal (Fuglie, 2001). The *Moringa* tree can be grown in a home garden, and it has the ability to with stand long‐term drought conditions (Ashifaq et al., 2012). The *Moringa* leaf powder can be stored at home for up to 6 months under recommended storage conditions (Beth & Lindsay, 2005); hence, the product can be accessed throughout the year even by poor families. The nutritional potential in *Moringa* leaf powder makes it an important ingredient in improving nutrient diversification in complementary food for children. Our study aimed to assess the impact of using *M. oleifera* leaf powder in complementary food to reduce anemia in children aged 6–24 months in Kisarawe District of Pwani region in Tanzania. The findings from this study are important in looking for solutions to address the high anemia prevalence among children in Tanzania and other affected low‐income countries.

## 2 METHODS

### 2.1 Study site

We conducted a community‐based intervention study in Kisarawe district of Pwani region between October 2014 and July 2015. The study involved four villages from two wards of Kibuta (Masanganya and Mhaga villages [intervention]) and Msimbu (Msimbu and Kitanga villages [control]) which were selected out of 15 available wards due to the high prevalence of children who had poor growth performance in these areas. This was based on routine statistics on monthly growth monitoring conducted by Community Health Workers (CHW) working with the health facilities which serve the communities. The children in the intervention community (Kibuta ward) were provided with *M. oleifera* leaf powder as a supplement to routine complementary food and nutrition education given to mothers/caretakers. In the control community (Msimbu ward), mothers/caretakers received only health education. Mothers/caretakers were advised to use at least 3 tablespoons (@ estimated 8 gm) or average of 25 g of *Moringa* leaf powder per day mixed in their child’s daily food (Beth & Lindsay, 2005). A total of 4 bottles of *Moringa* leaf powder with 200 gm each were supplied per child in a month.

### 2.2 Recruitment of study participants, measurements, and follow‐up

All children up to 2 years residing in the selected four villages from the two wards were invited for initial assessment to collect baseline information on age, sex, weight, height, and hemoglobin (Hb) levels. All measurements were taken to the nearest 0.1 unit, that is, 0.1 kg, 0.1 cm, and 0.1 g/dl for weight, height, and Hb level, respectively. Other child details recorded were history of any illness in the past 2 weeks, type of illness, and feeding practices including the use of micronutrient supplements and diversified food. We collected background information of parents/caretakers to include age, sex, occupation, level of education, economic activity, and size of the household. We also collected background characteristics of mothers/caretakers including age, sex, occupation, level of education, economic activity, and size of the household. This was done by using a semi‐structured questionnaire. Weight measurement was taken using Salter scale by hanging a child without clothing while length measurement was taken using a length board while the child is lying down (recumbent position)(WHO, 2008b). Hb levels were measured using a HemoCue Hb 201+ machine which gives quick and more precise results compared to other laboratory tests (Gwetu, Chhagan, & C. M. and K. S., 2014). All measurements were taken to the nearest 0.1 unit, that is, 0.1 kg, 0.1 cm, and 0.1 g/dl for weight, height, and Hb level, respectively. Other details recorded included history of any illness in the past 2 weeks, type of the disease a child suffered, and feeding practices including the use of micronutrient supplements and diversified food.

**Figure 1:**
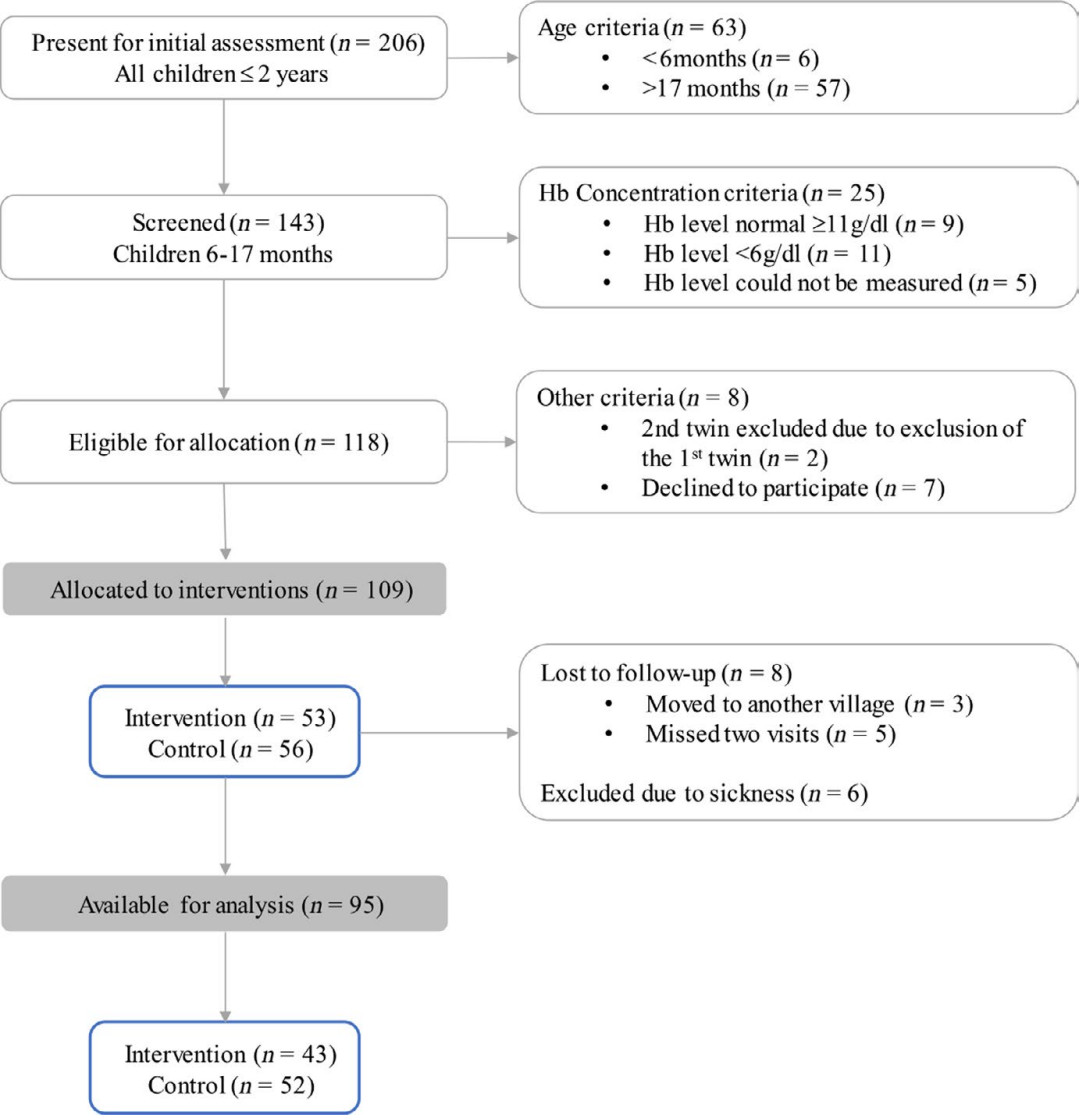
Flowchart of the study recruitment process

The project enrolled voluntarily anemic children (Hb <11 g/dl) of age between 6 months and 17 months from the study communities. Children found with Hb level <6 g/dl were excluded from the study and were referred to the nearest health facility for further appropriate management (Figure 1).

We conducted a monthly follow‐up on the children for a maximum period of 6 months in collaboration with study nurses from the nearby health facilities and CHWs. During monthly visits, the child’s age, height, weight, and history of illness were monitored. Hemoglobin levels were assessed after every 3 months in all study children.

A postintervention evaluation was done 3 months after suspending the interventions in order to assess long‐term effect of using Moringa leaf powder and the effect of withdrawing the intervention on child’s health and development. The evaluation involved assessment of Hb levels, focus group discussions (FGDs) with mothers/caretakers of the study children, and interviews with community health workers (CHWs) and nurses who participated in the study.

### 2.3 Data management, definition of key variables, and analysis

Baseline and follow‐up data were entered into EpiData software version 3.1 and later moved to STATA (Stata Corps, 2007) for analysis. Children were considered as having a normal Hb level when the value was ≥11 g/dl and anemia when a child had a Hb level <11 g/dl. Anemia levels were categorized as mild if Hb level was ≥10–≤11 g/dl; moderate between ≥7 and <10 g/dl and severe if the Hb level was <7 g/dl (WHO, 2011b). Descriptive statistics (mean and standard deviations, median and interquartile range (IQR), frequency distributions, and percentages) were done for all‐important variables such as sex, age, Hb concentration levels, anemia status, and distribution of the children in anemia categories. To measure and quantify the impact, the *absolute change* was calculated as the difference between the value at baseline and after 6 months of intervention, while the *percentage of effectiveness* was calculated as the ratio between the *absolute change* and the baseline value. For continuous variables, a paired *t* test was used to compare the mean Hb (within a group) and independent *t* test (between the control and intervention groups) at different time periods, while the test of proportions and chi‐square test were used to compare proportions, including anemia prevalence between different groups. Multivariable logistic regression analysis was used to identify factors associated with anemia among children. Odds ratios with 95% confidence interval (CI) were computed to assess the strength of association. Bivariate analysis between anemia status and each covariate was done to assess independent association of the factors. All variables with *p*‐value < 0.3 (in exception of age and sex) were added regardless of their bivariate association status) in the bivariate analysis. These were considered in multiple regression model to determine the effect of covariates on anemia while controlling for possible confounders. Statistical significance was considered at 5% level.

## 3 RESULTS

### 3.1 Characteristics of study participants

Initially, two hundred six (206) children were assessed, 49.5% (*n* = 102) from intervention community and 50.5% (*n* = 104) from control community. Men were 48.5%, and the overall mean age was 13.9 months (*SD* = 5.6). The mean Hb for all children was 8.7 g/dl (*SD* = 1.7), which was 8.4 g/dl (95% CI: 8.1–8.9) for intervention community and 8.9 g/dl (95% CI: 8.6–9.2) for control community. No significant difference was observed on children birthweights (median of 3.1, IQR: 3–3.5) or breastfeeding (over 75% of children still breastfed) between the two sites. Anemia prevalence was 91.4% (94.7% (95% CI: 90.3%–99.2%) intervention site; 88.3% (95% CI: 82.2%–94.5%) control site, *p*‐value = 0.1042). No significance difference was observed between the two sites on mothers/caretakers age, education, and size of households. A higher proportion of mothers/caretakers in intervention community were involved in agriculture (67.6%) than in the control site (44.2%).

The study enrolled a total of 109 children, of these 14 (12.8%) were dropped due to lost to follow‐up or sickness (Figure1). A total of 95 children were included in the final analysis, of which 54 (56.8%) were males and 41 (43.2%) females. The control community (Msimbu ward) had 52 children (27 males and 25 females) while the intervention community (Kibuta ward) had 43 children (27 males and 16 females). Children had an average age of 11.7 months (*SD* = 2.4) (Table 1). The mean Hb level at baseline was 8.1 g/dl (*SD* = 1.4) (intervention site: 8.3 g/dl [*SD* = 1.6]; control site: 7.9 g/dl [*SD* = 1.3], *p*‐value = 0.0943). Of those recruited, 8.7% had mild anemia, 71.7% had moderate, and 19.6% had severe anemia. The proportions of different levels of anemia were not significantly different between the two communities during the baseline period.

**Table 1:** Demographic characteristics of the study children

Variable	Control	Intervention	Total
Sex			
Males	27 (50%)	27 (50%)	54 (56.8%)
Females	25 (60.9%)	16 (39.1%)	41 (43.2%)
Total	52	43	95
Mean Age (*SD*) at first visit	11.8 (2.4)	11.6 (2.4)	11.7 (2.4)
Mean Hb level (*SD*) at first visit	7.9 (1.3)	8.3 (1.6)	8.1 (1.4)

### 3.2 Moringa leaf powder complementation

All families reported that they mixed *Moringa* leaf powder in babies’ porridge since this is the main meal for most infants in the study communities. In addition, some families reported mixing it in vegetable/bean stew (51.7%) and mashed banana/potato/cassava (13.8%). More than three‐quarters (79.3%) of the caretakers reported feeding their children *Moringa* mixed into food three times or more per day while others feeding at least twice. The main side effects reported in the first week of introducing *Moringa* leaf powder to the babies were loose stool (90.7%; *n* = 39), which persisted for an average of 3–4 days. In the first two months, most families reported feeding their babies half of the recommended amount of the Moringa leaf powder due to fear of the new product. During a focus group discussion, one participant said, *“in the first days we were afraid of giving our children a new product, but after realizing there is no harm and they are used to it, we started giving them more” (FGD Masanganya village).* However, none reported stopping usage. Feeding of *Moringa* leaf powder increased in the third month after improvements in children’s health were observed. After this, most families (93.1%) consumed three‐quarters or more of the supplied amount for the month. Before the study, most families (75.9%) were unaware of the nutritional benefit of *Moringa* tree although they had seen it growing in their localities.

### 3.3 Hemoglobin levels and anemia prevalence

**Figure 2:**
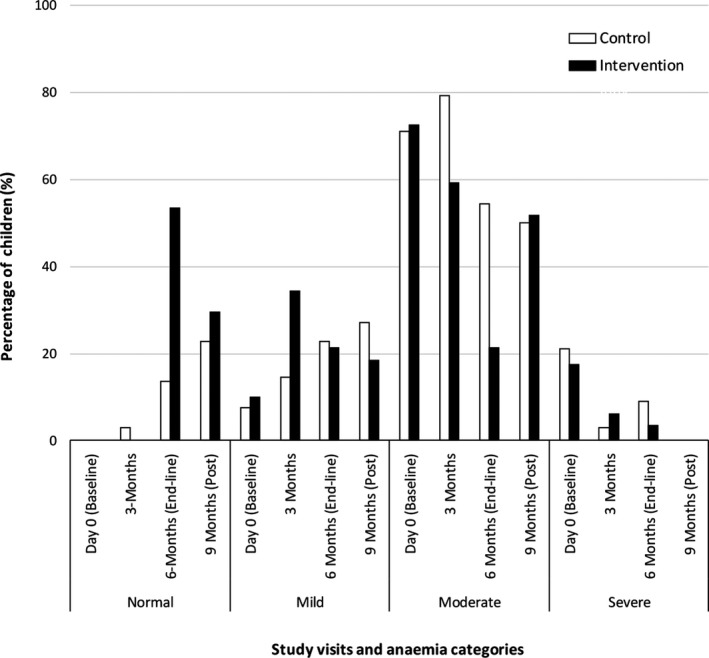
Pattern of anemia severity levels in the control and intervention communities during the study period

Table 2 depicts the status of Hb levels, anemia prevalence, and the proportion of children in different categories of anemia at baseline after 6 months of intervention, together with the absolute and percentage of effectiveness observed within the control and intervention sites. Changes in all anemia indicators were higher in the intervention communities than in the control communities, with the highest difference indicated in the mean Hb and the proportion of children with anemia (Table 2). After 6 months of observation, mean Hb level increased by 1.6 g/dl (95% CI: 1.4–1.7) in the control group while the increase in the intervention site was 2.6 g/dl (95% CI: 2.4–2.6). Anemia prevalence was reduced in the intervention communities by 53.6% (95% CI: 35.1, 72.0; *p* < 0.001) and in the control group by 13.6% (95% CI: 0.7, 28.0; *p* = 0.005). The prevalence of both moderate and severe anemia cases was also significantly reduced in the intervention communities as compared to the control communities (Table 2). After 6 months, the absolute changes in the severe cases of anemia were similar in the two sites (Table 2). A sharp decrease in the proportion of children with moderate anemia was observed after a continuing use (visits 2 and 3) of *M. oleifera* leaf powder (Figure 2).

**Table 2:** Status of anemia indicators 6 months after implementation

Site	Variable	Indicator			
		Mean Hb (g/dl) (95% CI)	% Anemia	% Moderate anemia	% Severe anemia
Control	At baseline	7.9 (7.5–8.2)	100	71.2	21.1
	After 6 months	9.4 (8.7–10.1)	86.4	54.6	9.1
	Absolute change	1.6 (1.4–1.7)	−13.6	−16.6	−12
	% Effectiveness	20.3	−13.6	−23.3	−56.9
Intervention	At baseline	8.3 (7.8–8.8)	100	67.4	16.3
	After 6 months	10.9 (10.2–11.4)	46.4	21.4	3.6
	Absolute change	2.6 (2.4–2.6)	−53.6	−46	−12.7
	% Effectiveness	31.3	−53.6	−68.2	−77.9
Significance testing (*p*‐value)	Within groups after 6 months	<0.0001	<0.0001	<0.0001	<0.0001
	Between groups after 6 months)	0.002*	0.004**	0.015**	0.415**

* *t* test.

** Two‐sample test of proportion.

**Figure 3:**
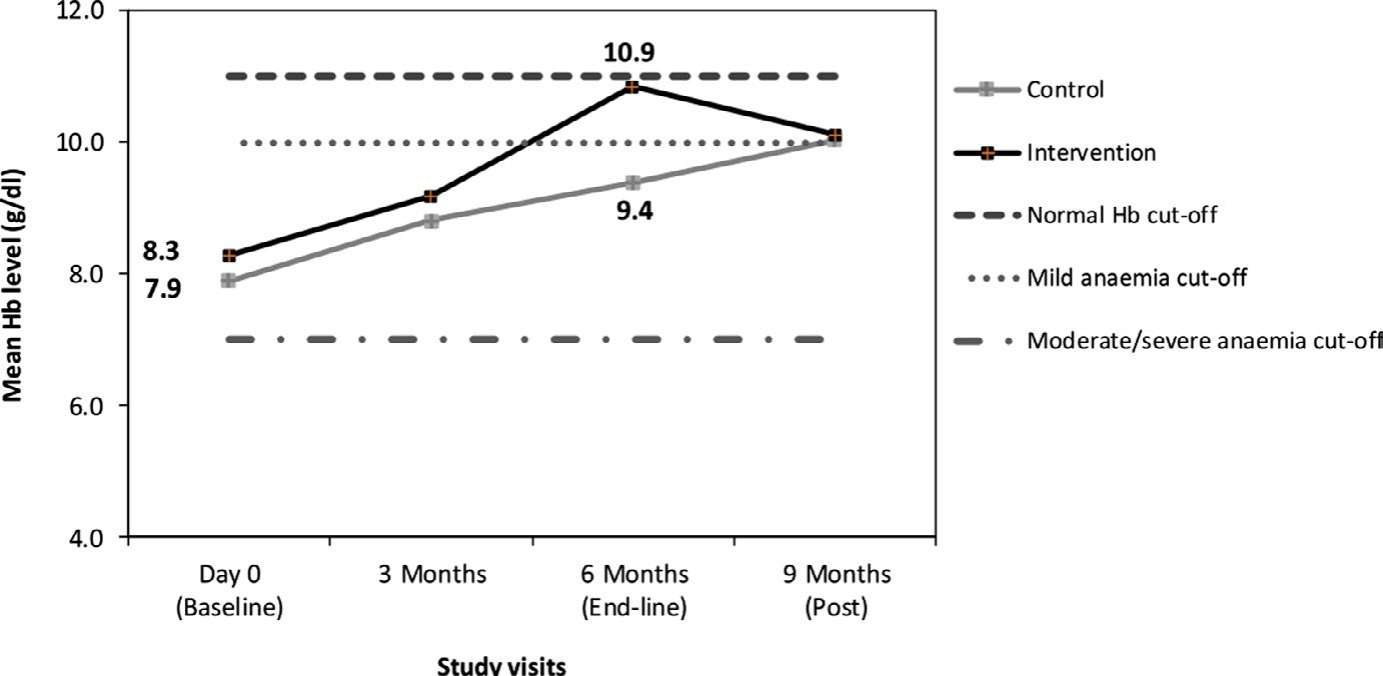
Changes in mean hemoglobin level in the control and intervention sites during intervention and postintervention period

Postintervention evaluation was done in 3 months after suspending the intervention, no cases of severe anemia were found, and the status for other severity levels was similar in the control and intervention communities: *p*‐value > 0.05 (Figure 2). However, a decrease in proportion of children with normal Hb and an increase in the proportion of children with moderate anemia in the intervention site were noted. A similar pattern was observed for the mean Hb levels (Figure 3).

The gap between the mean Hb levels of children from the control and the intervention communities was very similar during visits 1 and 2 (Figure 3). After continuous use of *Moringa* leaf powder, the gap widens and only a small proportion of children (~20%) from the intervention community remained in with moderate anemia while most (>75%) returned to mild and normal Hb levels, in contrary, those from the control community, over 56% where still with moderate anemia, although presenting a gradual, stable increase in mean Hb level (Figure 2, Figure 3). Three months after ceasing the provision of *M. oleifera* leaf powder, the mean Hb levels of children from both groups intersect at the mild anemia (Figure 3).

**Figure 4:**
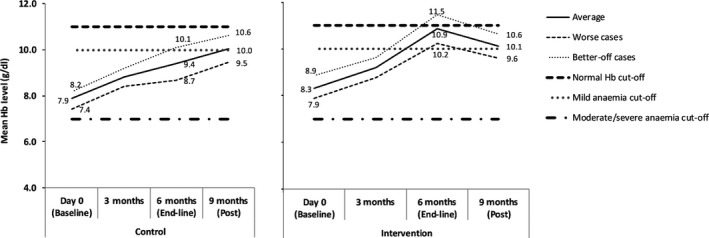
Pattern of mean hemoglobin levels in the intervention and control communities presenting in the worst and better‐off cases during the intervention period. (Lower limit of 95% CI taken as worst cases; upper limit as taken as better‐off cases; normal Hb = ≥11; mild anemia Hb = 10–≤11 g/dl); moderate (Hb: 7–≥10 g/dl); severe Hb = <7 g/dl)

We categorized children into better‐off cases (upper limit of the 95% CI of the mean Hb level) and worst cases (lower limit of the 95% CI of the mean Hb level) in order to assess the extended impact of the interventions. The patterns in control and intervention sites are illustrated in Figure 4.

After 6 months, better‐off cases from the intervention communities presented mean Hb level of 11.5 g/dl, while those from the control communities were performing similarly to worst cases in the intervention communities with average Hb of 10.1 g/dl (Figure 4). The worst cases from the control group were not able even to cross the mild anemia cutoff line. The continuing effect of the nutritional education is, however, demonstrated here as the both groups presented similar mean Hb levels even after no follow‐up was made.

Results of the multivariable regression analysis indicated that at a young age (6–18 months), using *M. oleifera leaf powder* and receiving nutritional education were associated with improvement in anemia as compared to older children (19–24 months) and receiving only nutritional education. The occurrence of malaria or diarrhea increased by over 4‐fold the odds of a child becoming anemic (Table 3).

**Table 3:** Factors associated with anemia among children aged 6–24 months involved in the *Moringa oleifera* study

Variable	Crude OR (95% CI)	Adjusted OR (95% CI)
Age (in months)		
6–18	0.23 (0.11,0.5)	0.17 (0.07,0.41)**
19–24	1.00	1.00
Sex		
Males	1.61 (0.76,3.43)	1.66 (0.74,3.74)
Female	1.00	1.00
Intervention		
*M. oleifera* + Nutritional education	0.35 (0.15,0.78)	0.23 (0.09,0.56)**
Nutritional education	1.00	1.00
Illness		
Malaria or diarrhea	1.93 (0.56,6.67)	4.4 (1.13,17.08)*
No illness	1.00	1.00

** *p*‐value < 0.001.

* *p*‐value < 0.05.

### 3.4 Other reported observed benefit of using moringa leaf powder

**Figure 5:**
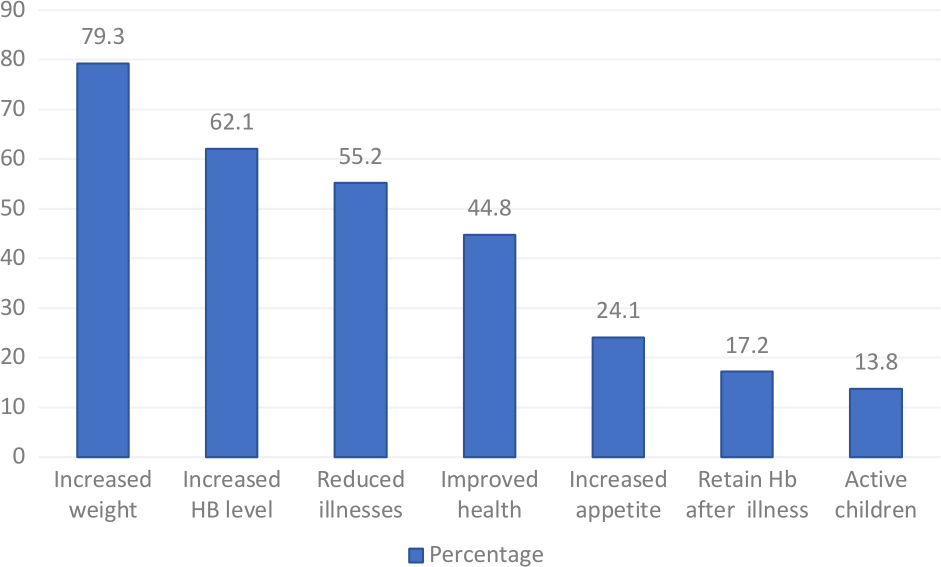
Tiff. Other reported benefit of using Moringa leaf powder

During the postintervention, evaluation in the intervention communities’ women who participated in the FGD and interviewed CHWs reported changes observed in their children who used the *Moringa* leaf powder, which included weight gain, increased Hb level, and reduced frequency of illness (Figure 5).

*We observed some changes, for instance, when a child gets sick, the Hb level was becoming very low, but when we used Moringa, the Hb level was OK (FGD, Muhaga village).*

*Frequency of illness has been reduced among project children compared those who were not in the project (CHW, Masanganya Village)*

*Children outside the project show no weight gain even after taking weight measurement for 3 consecutive months, but children involved in this project gained weight every month (CHW, Mhaga village).*

Awareness of Hb status and provision of nutrition education were reported to improve feeding practices in both the intervention and control communities. During focus group discussions, participants said;

*“After receiving nutritional counselling, I started feeding my child much more frequently. In the past I was just feeding him three times like an adult, but after being educated I started feeding him even five times in a day, giving him, some green vegetables and I saw his weight increasing” (FDG, Masanganya village)*.

*“Personally, this project has helped my child because before, her Hb was 6 but after being tested and given health education on best feeding practices, her Hb improved and reached 9….” (FDG, Msimbu village)*.

## 4 DISCUSSION

Dietary diversification within a minimum recommended number of meals is important to ensure adequate micronutrient supply (Thompson, 2011; WHO, 2008a; WHO & FAO, 2006), especially among poor communities who cannot access fortified processed food products. In this study, *M. oleifera*, leaf powder was used to diversify nutrient intake during complementary feeding by mixing it in a baby’s food due to its potential for a high composition of micronutrients. Feeding of *Moringa‐*mixed food was carried out at least three times a day, and this frequency remained the same during the project period. This feeding frequency of three meals per day is in line with the WHO recommendation for complementary feeding for infants and young children who are still on breast feeding (WHO, 2008a). The project contributed to improvement in feeding habit in the study communities, above the national average of 40% in children aged 6–23 months (MoHCDGEC, NBS, OCGS, & ICF, 2016). The reported low intake of *Moringa* leaf powder in the first 2 months is quite acceptable since adaptation to new food products takes time. Moreover, demand of a product usually increases as consumers experience the benefit of it; hence, consumption of *Moringa* was reported to improve after observed health improvement in children. No adverse side effects from the consumption of *Moringa* leaf powder were reported, and similar findings were reported in a study conducted in Senegal (Fuglie, 2001).

Anemia was significantly reduced when using *Moringa* leaf powder (Table 2) for a longer period of time. The amount of *Moringa* consumed is also significant, since most mothers reported increasing the amount of *Moringa* they used in the third month after observing an improvement in their baby’s health, which resulted to a sharp increase in Hb level afterward (Figure 3). Other studies conducted in India and Indonesia found a significant increase in Hb level among women who used *M. Oleifera* leaf extract (Sindhu, Mangala, & Sherry, 2013; Suzana et al., 2017). Moreover, the high content of vitamin A present in *Moringa* is an added advantage that contributed to the considerable reduction of anemia. The study in India found beta‐carotene from consumption of Moringa for 1 month had a protective effect on iron availability which resulted in 10% anemia reduction from moderate to mild level (Nambiar, Patel, Gosai, Nithya, & Desai, 2012). The argument is supported by a study conducted in Indonesia that found iron supplementation when combined with vitamin A supplement, resulted in a higher reduction of anemia (98%) than from just having iron supplementation alone (68%) (Suharno et al., 1993). Likewise, the study in Ethiopia found children who received vitamin A supplement had higher Hb level than those who did not (Gebremedhin, 2014). Similarly, more health benefits from using *Moringa* were reported by people who used it as part of their routine diet (Fuglie, 2001). Similarly, more health benefits from using *Moringa* were reported by people who used it as part of their routine diet (Fuglie, 2001).

The use of *Moringa* was found to be very powerful in tackling moderate anemia, as opposed to severe anemia cases. The reduction of moderate anemia in the intervention communities was three times more than in the control communities. The reduction in severe anemia cases was not significantly different between the two groups. One explanation for this is that severely anemic children might have other complications and deficiencies, which could hinder quick improvement. However, at the end of the intervention, there were no severe anemia cases seen in the study children, which might suggest an improvement in feeding knowledge and practice, but also the effect of close Hb monitoring and continued provision of nutritional education. This finding is also supported by the fact that iron absorption is stimulated by exhausting of body iron reserve and the process of red blood cell production (Milman, 2006; Thompson, 2011).

Furthermore, weight gain was reportedly observed in children who used *Moringa* leaf powder. Similar findings of weight gain in children who used *Moringa* leaf powder were reported in Senegal (Mahmood et al., 2010), India (Srikanth, 2014), and Burkina Faso (Zongo, Zoungrana, Savadogo, & Traoré, 2013). Weight gain may be attributed to high composition of digestible protein in *Moringa* that is important for body growth (Ashifaq et al., 2012; Fahey, 2005; Liyanage et al., 2014; Mahmood et al., 2010). Moreover, women reported a reduction in frequency of illnesses in their children after using *Moringa* leaf powder. Reduction of illnesses may be attributed to improved body immunity, since the use of *Moringa* powder supplemented tea in school children in Nigeria was found to increase white blood cell which is important for the body defense against infections (Nurain, 2015). Furthermore, *Moringa* has additional benefits in disease prevention and treatment due to its medicinal properties such as antimicrobial and anti‐inflammatory properties (Ashifaq et al., 2012; Babu, 2000; Daba, 2016; Faizal et al., 2014; Kasolo, Bimenya, Ojok, Ochieng, & Ogwal‐okeng, 2010). This improvement in children health supports the potential of *M. oleifera* leaf powder in reducing multiple malnutrition problems.

According to the global strategy for infant and young children feeding, consistency and complete information about appropriate food and feeding practices is a key determinant of undernutrition, rather than a lack of food (WHO, 2003). In this study, the value of consistent health education and close monitoring of Hb status was observed. Despite the control community being offered health education alone, they retained a gradual and consistent improvement in anemia levels (Figure 2). Additionally, children were tested for their anemia status on a quarterly basis; hence, awareness of a child’s Hb status encouraged mothers to improve feeding practices.

Majority of the participants had low awareness of the nutritional potential of *M. oleifera* leaf powder. Even in other countries, where *Moringa* has been used for years for healing purposes for years, its nutritional benefit is not well known to the wide range of population. According to a report by Fahey (2005), even in places where *Moringa* has been used for many years such as India and some West African countries, most of the promotion of *Moringa* benefits are only focused on its medicinal benefits. Despite there being a lot of scientific evidence (Arise, Arise, Sanusi, Esan, & Oyeyinka, 2014; Babu, 2000; Busani et al., 2011; Oyeyinka & Oyeyinka, 2018) and feeding studies which have reported the nutritional potential of *Moringa* (Nambiar et al., 2012; Nurain, 2015; Srikanth, 2014; Suzana et al., 2017; Zongo et al., 2013), this knowledge is not tapped into poor countries that are highly affected by malnutrition. Therefore, sharing of best practices and study findings should not end in scientific, academic platforms such as journals and books, but more efforts should be invested to spread the information to the general public.

## 5 STUDY LIMITATION

The feeding of *Moringa* leaf powder in the first 2 months was reported to be half of the recommended amount and was increased up to three‐quarters after the third month due to a fear of the new product. The health improvements seen in children after they had been taking *Moringa* for a longer period encouraged mothers to increase the amounting of *Moringa* leaf powder when feeding their babies.

## 6 CONCLUSIONS

High prevalence of iron deficiency anemia among children under 2 years is a public health concern and has an irreversible impact on intellectual development, future learning, and earning capacity. The use of *Moringa* leaf powder significantly reduced the prevalence of anemia cases by half and worked better in moderate anemia cases. The health benefits of consistent use of the recommended amount of *M. oleifera* leaf powder over a longer period are clearly presented. Promoting the long‐term use of *M. oleifera* leaf powder, with a clear information about dosage, should be taken as an alternative solution for addressing anemia and other nutrition deficiencies in Tanzania.

Dietary diversification by using high‐density nutritional locally available food is still a long‐term and sustainable solution to iron deficiency anemia and other nutritional deficiencies. Research on dietary diversification and food fortification should look at utilizing the nutritional potential of *M. oleifera* leaf powder to address malnutrition problem in poor countries.

## CONFLICT OF INTEREST

The authors declare that they have no competing interests.

## ETHICAL APPROVAL

This study conforms to the Declaration of Helsinki. The study was approved by the National Health Research Ethical sub‐committee of the Medical Research Coordinating Committee, certificate number NIMR/HQ/R8a/VolIX/1754.

## CONSENT FOR PUBLICATION

The National Institute for Medical Research gave permission for publication of this manuscript.

## INFORMED CONSENT

Written informed consent was obtained from all study participants.

## Data Availability

Dataset analyzed for this study is available from the corresponding author on reasonable request.
